# New records of Chrysochroinae Laporte de Castelnau, 1835 (Coleoptera, Buprestidae) from China

**DOI:** 10.3897/BDJ.12.e115599

**Published:** 2024-03-11

**Authors:** Hong-Mu Ai, Zhi-Hao Qi, Rong-Xiang Su, Zhi-Yu Liao, Hai-Tian Song

**Affiliations:** 1 College of Plant Protection, Fujian Agriculture and Forestry University, Fuzhou, China College of Plant Protection, Fujian Agriculture and Forestry University Fuzhou China; 2 Fujian Academy of Forestry, Fuzhou, China Fujian Academy of Forestry Fuzhou China

**Keywords:** jewel beetle, *Cardiaspis*, *Chrysochroa*, *Demochroa*, *Xanthocata*, new record, south China

## Abstract

**Background:**

Chrysochroinae Laporte de Castelnau, 1835 is the very colourful subfamily of Buprestidae. There are 127 species and subspecies of the subfamily which have been recorded in China.

**New information:**

In this paper, we reported three genera, two subgenera and five species of the subfamily Chrysochroinae Laporte de Castelnau, 1835 (Coleoptera, Buprestidae) which are all newly recorded from China. These reported taxa belong to two tribes and four genera: Chrysochroa (Chroodema) corbetti (Kerremans, 1893), Chrysochroa (Pyranthe) fulgens ephippigera White, 1843, Demochroa (Demoxantha) gratiosa indica Csiki, 1900, *Xanthocatabonvouloirii* (Deyrolle, 1861) (all the above four being Chrysochroini) and *Cardiaspismouhotii* E. Saunders, 1866 (Dicercini). The five newly-recorded species are briefly described, illustrated and supplemented with relevant biological information.

## Introduction

Chrysochroinae Laporte de Castelnau, 1835 is the very colourful subfamily of Buprestidae, with many large ornamental species living in tropical rainforests. Before this study, 127 species and subspecies of the subfamily have been recorded in China, belonging to five tribes and 17 genera (Bellamy 2008a, Bellamy 2008b, Kubáň 2016, Ong and Hattori 2019, Frank and Sekerka 2020, Peng et al. 2021, Frank 2022, Peng and Song 2022, Qi et al. 2022, Hołyński 2023, Ong et al. 2023).

Five newly-recorded species of the subfamily Chrysochroinae from China are presented herein: Chrysochroa (Chroodema) corbetti (Kerremans, 1893) found in Hainan Province, Chrysochroa (Pyranthe) fulgens ephippigera White, 1843, Demochroa (Demoxantha) gratiosa indica Csiki, 1900, *Xanthocatabonvouloirii* (Deyrolle, 1861) and *Cardiaspismouhotii* E. Saunders, 1866 found in Yunnan Province. The five species are briefly described, illustrated and supplemented with relevant biological information.

## Materials and methods

**Figure 1.**
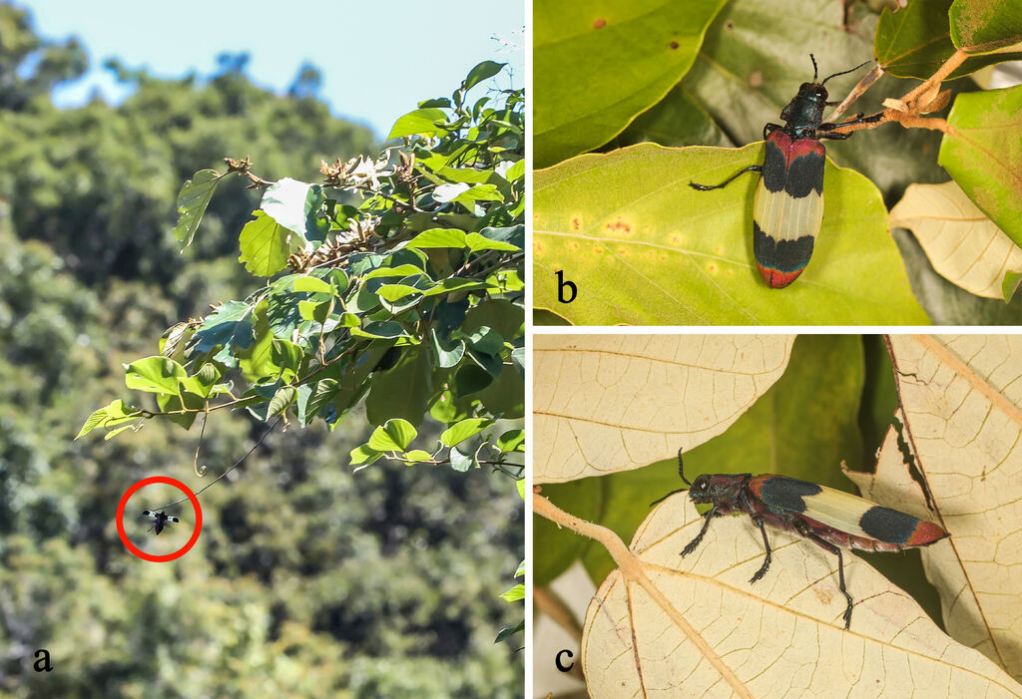
Male living individuals of Chrysochroa (Chroodema) corbetti (Kerremans, 1893) from Sanya, Hainan: **a** an individual flying around the host plant *Pterospermumacerifolium*; **b** dorsal view; **c** lateral view of feeding *P.acerifolium* leaves.

Habitus photographs were taken using a Canon 5D mark IV digital camera with a Canon EF 100 mm USM macrolens. A Canon MT-26EX twin flash was used as the light source. Fig. 1A was taken using a Canon 5D mark IV digital camera with a Canon EF 70-200 mm f/2.8L IS III USM lens. Photographs of smaller characters were photographed using a Keyence VHX-5000 digital microscope with a Keyence VH-Z20R zoom lens (20-200×). The photograph in Fig. 4 was taken using a Canon EOS 50D by Lu Qiu, while that in Fig. 7 was taken using an iPhone 14 smartphone by Yi-Wei Wang. The images were processed and combined into figures using Adobe Photoshop CC 2019. All photographs were taken by the authors, except those with captions.

Abbreviations for collections in this study are:

- **CHTS** Collection of Hai-Tian Song, Fuzhou, China.
- **KIZ** Kunming Institute of Zoology, Chinese Academy of Sciences, Kunming, China.

## Checklists

### Taxon treatments

#### Buprestidae

Leach, 1815

58B4D0C9-F595-5E36-8D55-9E65E93CC8F4

#### Chrysochroinae

Laporte de Castelnau, 1835

A1A2C6BC-68C2-5026-B9F1-C66CB424804D

#### Chrysochroini

Laporte de Castelnau, 1835

E9974AE0-292F-56FE-84F9-E0652725A237

#### Chrysochroa

Dejean, 1833

FA92C1BE-F332-5B6C-92A0-51DEA9AA214B

#### Chroodema

Hołyński, 2009

DB3AF643-94A0-54E6-99C6-420A96915D75

##### Notes

New record of China.

#### Chrysochroa (Chroodema) corbetti

(Kerremans, 1893)

EAF60C4F-ADD9-5360-BFB4-D4A8F1718A08

Demochroa corbetti Kerremans, 1893: 326. Kerremans 1893

##### Materials

- **Type status:** Other material. **Occurrence:** individualCount: 1; sex: male; lifeStage: adult; occurrenceID: B2DA381F-62CC-5534-8554-9813F326C55E; **Location:** country: China; stateProvince: Hainan; verbatimElevation: 350 m; locationRemarks: label: Hainan, Sanya City, Mt. Lizhiling, 28.06.2020, Jia-Sheng Lu Leg.; verbatimCoordinates: 18°21'N 109°27'E; **Identification:** identifiedBy: Hai-Tian Song; dateIdentified: 2020; **Event:** samplingProtocol: sweep net; habitat: Rain Forest; **Record Level:** institutionCode: CHTS; basisOfRecord: PreservedSpecimen
- **Type status:** Other material. **Occurrence:** individualCount: 1; sex: male; lifeStage: adult; occurrenceID: 9B6935EC-B048-5FC0-A52E-02E8BB48EB79; **Location:** country: China; stateProvince: Hainan; verbatimElevation: 390 m; locationRemarks: label: Hainan, Sanya City, Mt. Lizhiling, 15.06.2021, Jia-Sheng Lu Leg.; verbatimCoordinates: 18°21'N 109°27'E; **Identification:** identifiedBy: Hai-Tian Song; dateIdentified: 2021; **Event:** samplingProtocol: sweep net; habitat: Rain Forest; **Record Level:** institutionCode: CHTS; basisOfRecord: PreservedSpecimen
- **Type status:** Other material. **Occurrence:** individualCount: 2; sex: 2 males; lifeStage: adult; occurrenceID: 45F3671A-6C00-5AD6-93CB-EEB86190EA3D; **Location:** country: China; stateProvince: Hainan; verbatimElevation: 390 m; locationRemarks: label: Hainan, Sanya City, Mt. Lizhiling, 19.06.2021, Hai-Tian Song & Zhi-Hao Qi Leg.; verbatimCoordinates: 18°21'N 109°27'E; **Identification:** identifiedBy: Hai-Tian Song; dateIdentified: 2021; **Event:** samplingProtocol: high sweep net; habitat: Rain Forest; **Record Level:** institutionCode: CHTS; basisOfRecord: PreservedSpecimen
- **Type status:** Other material. **Occurrence:** individualCount: 1; sex: male; lifeStage: adult; occurrenceID: A1130627-711B-5D53-83E7-3C5504167273; **Location:** country: China; stateProvince: Hainan; verbatimElevation: 390 m; locationRemarks: label: Hainan, Sanya City, Mt. Lizhiling, 06.2023, Jia-Sheng Lu Leg.; verbatimCoordinates: 18°21'N 109°27'E; **Identification:** identifiedBy: Hai-Tian Song; dateIdentified: 2023; **Event:** samplingProtocol: sweep net; habitat: Rain Forest; **Record Level:** institutionCode: CHTS; basisOfRecord: PreservedSpecimen
- **Type status:** Other material. **Occurrence:** individualCount: 1; sex: unknown; lifeStage: adult; occurrenceID: 01D43CAA-C80D-561D-807B-BFF69BF348D9; **Location:** country: China; stateProvince: Hainan; locationRemarks: label: Hainan, Ledong County, Jianfengling National Forest Park, Tianchi, 02.05.2022, local collector Leg.; **Identification:** identifiedBy: Hai-Tian Song; dateIdentified: 2022; **Event:** habitat: Rain Forest; **Record Level:** institutionCode: CHTS; basisOfRecord: PreservedSpecimen

##### Host of

*Pterospermumacerifolium* (quote from Ek-Amnuay (2008)), *P.heterophyllum* (Fig. 1, new host plant found in Hainan).

##### Distribution

China (Hainan) (new country record); Myanmar; Vietnam; Thailand (Akiyama and Ohmomo 2000, Bellamy 2008a, Ek-Amnuay 2008).

##### Notes

The adult has been found in Sanya, Hainan Province with *Catoxanthabrunneahainana* (Kurosawa, 1991) feeding together on *Pterospermumheterophyllum* leaves.

**Figure 2.**
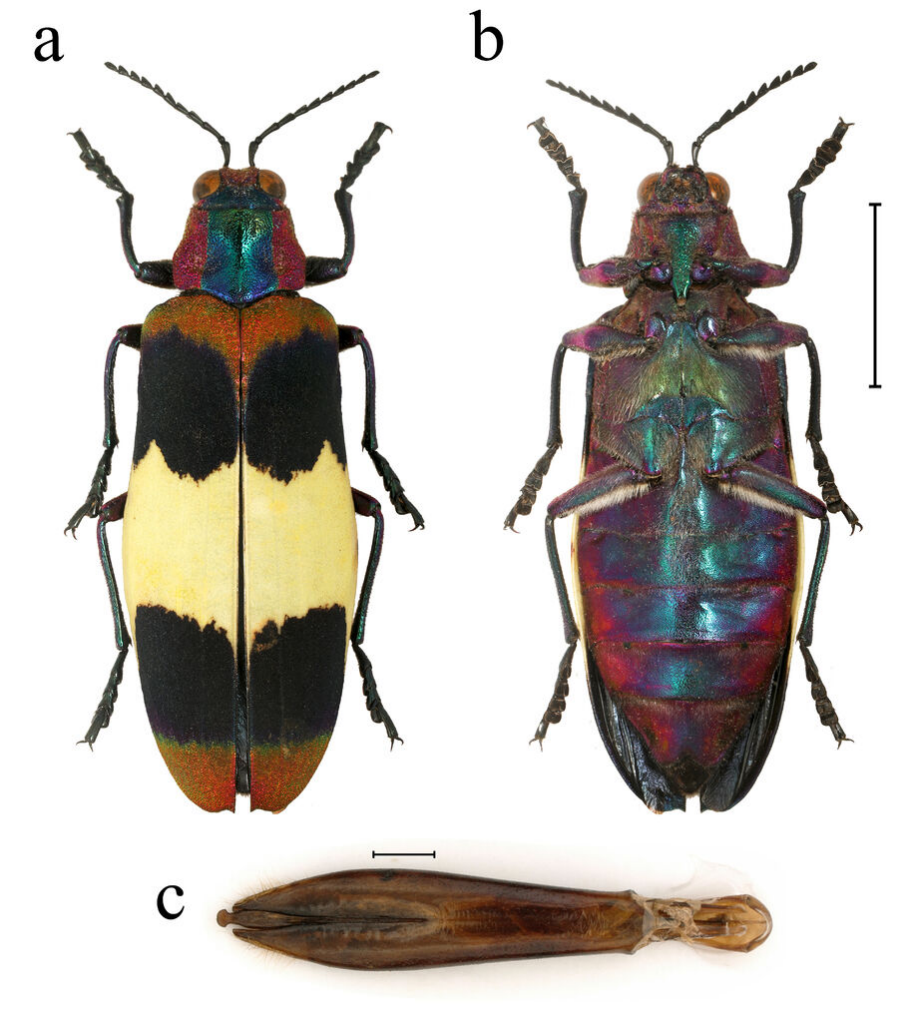
Chrysochroa (Chroodema) corbetti (Kerremans, 1893), male, from Sanya, Hainan Province: **a** dorsal view of habitus; **b** ventral view of habitus; **c** dorsal view of aedeagus. Scale bars: a, b = 10.0 mm; c = 1.0 mm.

**Description**:

**Male** (Fig. 2): Body (n = 4) length: 33.3–36.8 mm, width: 12.1–13.5 mm, length/width ratio: 2.60–2.94. Body polychromatic; head, pronotum and base of elytra densely punctate. Head bicoloured, frons and both sides of posterior margin metallic purple, other areas metallic blue; antenna black with 11 antennomeres, antennomeres V–XI expanded. Pronotum tricoloured, metallic purple-red at sides, with a broad metallic green or blue stripe at median, more than half of pronotum; narrow in front and broader behind, widest at basal third. Scutellum absent. Elytra four-coloured, bearing two large blue to black bands at the front half and behind half of each elytron, their borders reaching the suture and the lateral margins, with metallic yellow-orange bands and metallic-greenish suffusion at base and apex and a broad creamy-yellow band at middle; lateral margins of elytra smooth, non-serrated, with one small spine at the apex of each elytron, widest at nearly half of elytra. All tibiae and femora metallic blue to purple-red; outer margin at apex of protibia with nearly triangular protrusion. Abdominal sternum and ventrites metallic blue to purple, ventrite V triangularly excavated at tip. The characters of the male genitalia are shown in Fig. 2.

**Female**: not examined.

#### Pyranthe

Gistel, 1834

C087A4E1-CCD7-5963-A1F9-B83432C93140

#### Chrysochroa (Pyranthe) fulgens ephippigera

White, 1843

3158F457-1E5D-5E65-A504-D9D484F4ACD2

Chrysochroa ocellata var. ephippigera White, 1843: 343. White 1843

##### Materials

- **Type status:** Other material. **Occurrence:** individualCount: 1; sex: male; lifeStage: adult; occurrenceID: 6DC302D9-D8C1-5126-BE64-784C1F5E6CFE; **Location:** country: China; stateProvince: Yunnan; verbatimElevation: 1006 m; locationRemarks: label: Yunnan, Dehong Dai and Jingpo Autonomous Prefecture, Yingjiang County, Nabang Town, Rongshuwang, 06.2020, local collector Leg.; verbatimCoordinates: 24°39'N 97°36'E; **Identification:** identifiedBy: Hai-Tian Song; dateIdentified: 2020; **Event:** habitat: Forest; **Record Level:** institutionCode: CHTS; basisOfRecord: PreservedSpecimen
- **Type status:** Other material. **Occurrence:** individualCount: 1; sex: unknown; lifeStage: adult; occurrenceID: E0D1BB9C-6D12-540D-AEC9-E7E325BB27BA; **Location:** country: China; stateProvince: Yunnan; locationRemarks: label: Yunnan, Dehong Dai and Jingpo Autonomous Prefecture, Yingjiang County, Xima Town, 12.2020, local collector Leg.; **Identification:** identifiedBy: Hai-Tian Song; dateIdentified: 2021; **Event:** habitat: Forest; **Record Level:** institutionCode: CHTS; basisOfRecord: PreservedSpecimen
- **Type status:** Other material. **Occurrence:** individualCount: 1; sex: female; lifeStage: adult; occurrenceID: 3C25E79A-CC5A-59DD-99C1-EA6B9E103530; **Location:** country: China; stateProvince: Yunnan; locationRemarks: label: Yunnan, Dehong Dai and Jingpo Autonomous Prefecture, Yingjiang County, Xima Town, 08.2021, local collector Leg.; **Identification:** identifiedBy: Hai-Tian Song; dateIdentified: 2021; **Event:** habitat: Forest; **Record Level:** institutionCode: CHTS; basisOfRecord: PreservedSpecimen
- **Type status:** Other material. **Occurrence:** individualCount: 2; sex: 2 females; lifeStage: adult; occurrenceID: 7D7B7DB4-972A-5BB3-AB41-8901AA1C7782; **Location:** country: China; stateProvince: Yunnan; locationRemarks: label: Yunnan, Dehong Dai and Jingpo Autonomous Prefecture, Yingjiang County, Xima Town, 10.12022, local collector Leg.; **Identification:** identifiedBy: Hai-Tian Song; dateIdentified: 2023; **Event:** habitat: Forest; **Record Level:** institutionCode: CHTS; basisOfRecord: PreservedSpecimen

##### Host of

*Cratoxylon* sp. (quote from Ek-Amnuay (2008)).

##### Distribution

China (Yunnan) (new country record); Myanmar; Laos; Vietnam; India; Thailand (Akiyama and Ohmomo 2000, Ek-Amnuay 2008, Hołyński 2009).

##### Notes

All individuals found in China with tibiae and femora metallic purple-red.

**Figure 3.**
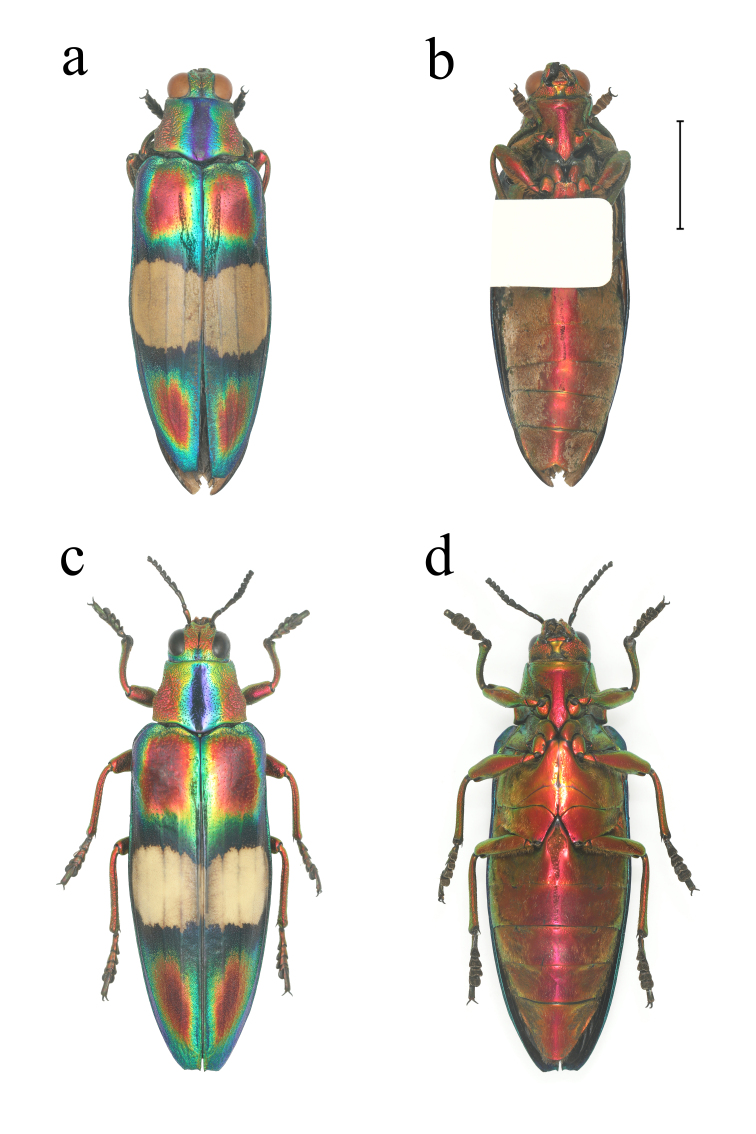
Chrysochroa (Pyranthe) ephippigera (White, 1843) from Yingjiang, Yunnan: **a** male dorsal view; **b** male ventral view; **c** female dorsal view; **d** female ventral view. Scale bar = 10.0 mm.

**Description**:

**Male** (Fig. 3): Body (n = 1) length: 37.4 mm, width: 13.0 mm, length/width ratio: 2.88. Body polychromatic and shiny; head, pronotum and elytra densely punctate. Head tricoloured, metallic green or blue, tinted with coppery-red at middle of frons. Pronotum five-coloured, metallic purple-red at sides with a broad longitudinal dark blue stripe at median, with transition areas of metallic blue, green and orange between the two colours; narrow in front and broader behind, widest at base. Scutellum absent. Elytra five-coloured, the basal and apical parts tinted with two large metallic purple-red spots on each elytron, with a narrow metallic orange ring around each spot on the periphery, followed by a wide metallic light blue ring around; a broad creamy-yellow patch across the middle, with two narrow dark blue bands in front and behind the creamy-yellow patch; lateral margins of elytra smooth, non-serrated, with one small spine at apex of each elytron, widest at nearly half of elytra. All tibiae and femora metallic purple-red; outer margin at apex of protibia with nearly triangular protrusion. Abdominal sternum and ventrites metallic yellow-orange to purple, with dense setae, ventrite V triangularly excavated at tip.

**Female** (Fig. 3): Body (n = 3) length: 39.4–41.6 mm, width: 13.2–14.0 mm, length/width ratio: 2.85–3.08. The female specimen differs from the male in the colour spots. Setae on the ventral side of body more sparse. Ventrite V without triangular excavation at tip. Other morphological descriptions refer to male specimens.

#### Demochroa

White, 1859

E7C9F737-8F79-5EC3-8E88-02E5664240F1

##### Notes

New record of China.

#### Demoxantha

Hołyński, 2009

B3D5B655-E108-55E7-89AC-D76150BB7DAA

##### Notes

New record of China.

#### Demochroa (Demoxantha) gratiosa indica

Csiki, 1900

046E9131-40B0-5D1D-911F-ED9C5EEC88B9

Demochroa indica Csiki, 1900: 402. Csiki 1900
= *Demochroabowringii* Waterhouse, 1904: 265. Waterhouse 1904
= *Demochroameldolana* Waterhouse, 1904: 265. Waterhouse 1904

##### Materials

- **Type status:** Other material. **Occurrence:** individualCount: 1; sex: unknown; lifeStage: adult; occurrenceID: BEDA431B-B565-5F03-806A-5BA1582EC861; **Location:** country: China; stateProvince: Yunnan; verbatimElevation: 200-400 m; locationRemarks: label: Yunnan, Dehong Dai and Jingpo Autonomous Prefecture, Yingjiang County, Nabang Town, 03.06.2018, photo by Lu Qiu.; **Identification:** identifiedBy: Hai-Tian Song; dateIdentified: 2018; **Event:** habitat: Forest; **Record Level:** basisOfRecord: HumanObservation

##### Distribution

China (Yunnan) (new country record); Bangladesh; India; Laos; Thailand (Akiyama and Ohmomo 2000, Ek-Amnuay 2008, Hołyński 2009, Kubáň 2016).

##### Notes

The purple part is replaced by light green in most specimens of the species. Since the first individual found in China was incomplete, we provisionally classified it as the subspecies indica based on the subspecies distribution map of Hołyński (2009).

**Figure 4.**
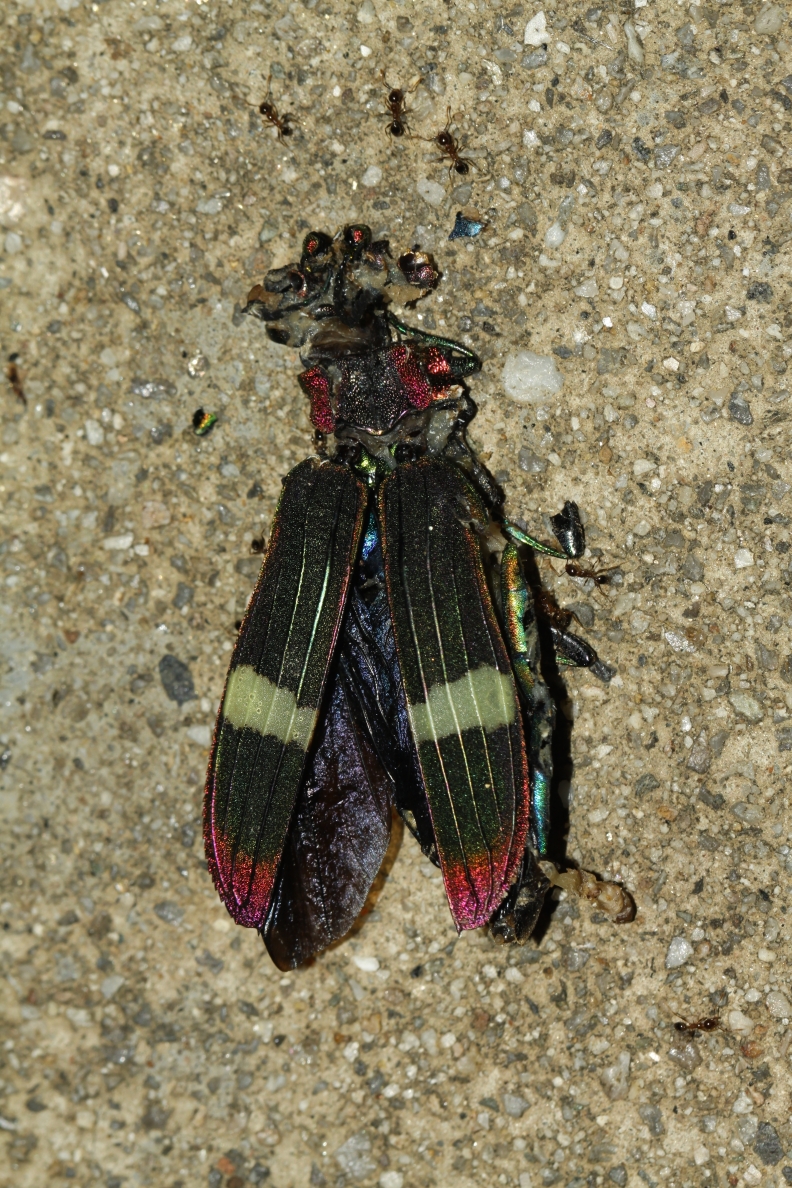
A crushed individual of Demochroa (Demoxantha) gratiosa Deyrolle, 1864 from Yingjiang, Yunnan Province; the photo taken by Lu Qiu in Nabang Town on 3 June 2018.

**Description**:

Sex unknown (Fig. 4): Body trichromatic and shiny. Pronotum transverse, bicoloured, dark green with two purple longitudinal stripes on lateral margins; anterior margin nearly straight; lateral margins concave at half of the base; posterior margin bisinuate. Scutellum not distinct. Elytra tricoloured, dark green with two large, transverse, white stripes behind the middle, terminal areas purple; widest behind half of the elytra, each elytron with four convex costae; apical half of lateral margins serrated, apices of elytra nearly truncate with one spine at apex of each elytron. All tibiae metallic green.

#### Xanthocata

Kubáň, 2016

26D7BFCD-9FCC-5AB3-82D9-E77C53AEDC53

##### Notes

New record of China.

#### Xanthocata bonvouloirii

(Deyrolle, 1861)

DA097FD5-F028-521A-9172-996684197EAE

Catoxantha bonvouloirii Deyrolle, 1861: 395. Deyrolle 1861
= *Catoxantharegina* Schaufuss, 1863: 168. Schaufuss 1863

##### Materials

- **Type status:** Other material. **Occurrence:** individualCount: 1; sex: female; lifeStage: adult; occurrenceID: 731ECCD6-862C-5689-BA43-A680BF1C8741; **Location:** country: China; stateProvince: Yunnan; verbatimElevation: 1000 m; locationRemarks: label: Yunnan, Dehong Dai and Jingpo Autonomous Prefecture, Yingjiang County, Xima Town, 10.2020, local collector Leg.; **Identification:** identifiedBy: Hai-Tian Song; dateIdentified: 2022; **Event:** habitat: Forest; **Record Level:** institutionCode: CHTS; basisOfRecord: PreservedSpecimen
- **Type status:** Other material. **Occurrence:** individualCount: 3; sex: 1 male, 2 females; lifeStage: adult; occurrenceID: 2DF2B98C-7C36-56D0-AF8A-1E5847989F71; **Location:** country: China; stateProvince: Yunnan; verbatimElevation: 760 m; locationRemarks: label: Yunnan, Dehong Dai and Jingpo Autonomous Prefecture, Yingjiang County, Nongzhang Town, 08.04.2023, local collector Leg.; **Identification:** identifiedBy: Hai-Tian Song; dateIdentified: 2023; **Event:** habitat: Forest; **Record Level:** institutionCode: CHTS; basisOfRecord: PreservedSpecimen

##### Distribution

China (Yunnan) (new country record); India; Bhutan; Myanmar; Thailand; Laos; Vietnam; Indonesia (Akiyama and Ohmomo 2000, Bellamy 2008a, Kubáň 2016).

##### Notes

**Figure 5.**
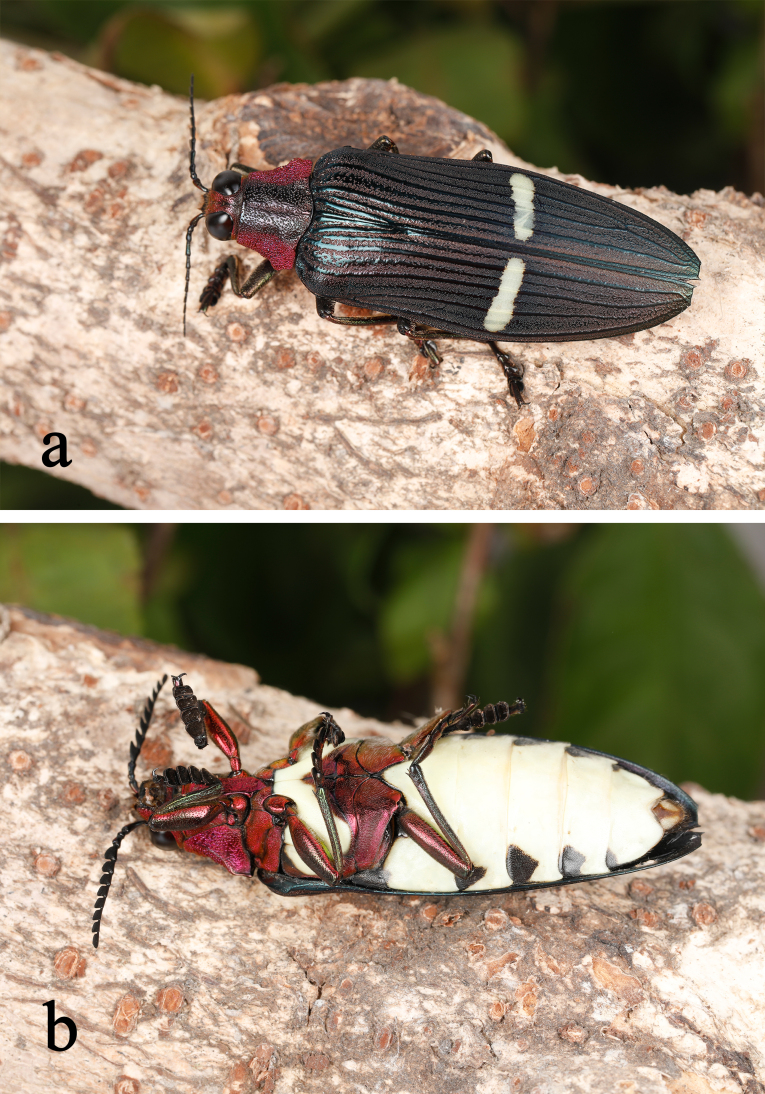
A male living individual of *Xanthocatabonvouloirii* (Deyrolle, 1861) from Yingjiang, Yunnan Province: **a** dorsal view; **b** ventral view.

The living individual is shown in Fig. 5.

**Figure 6.**
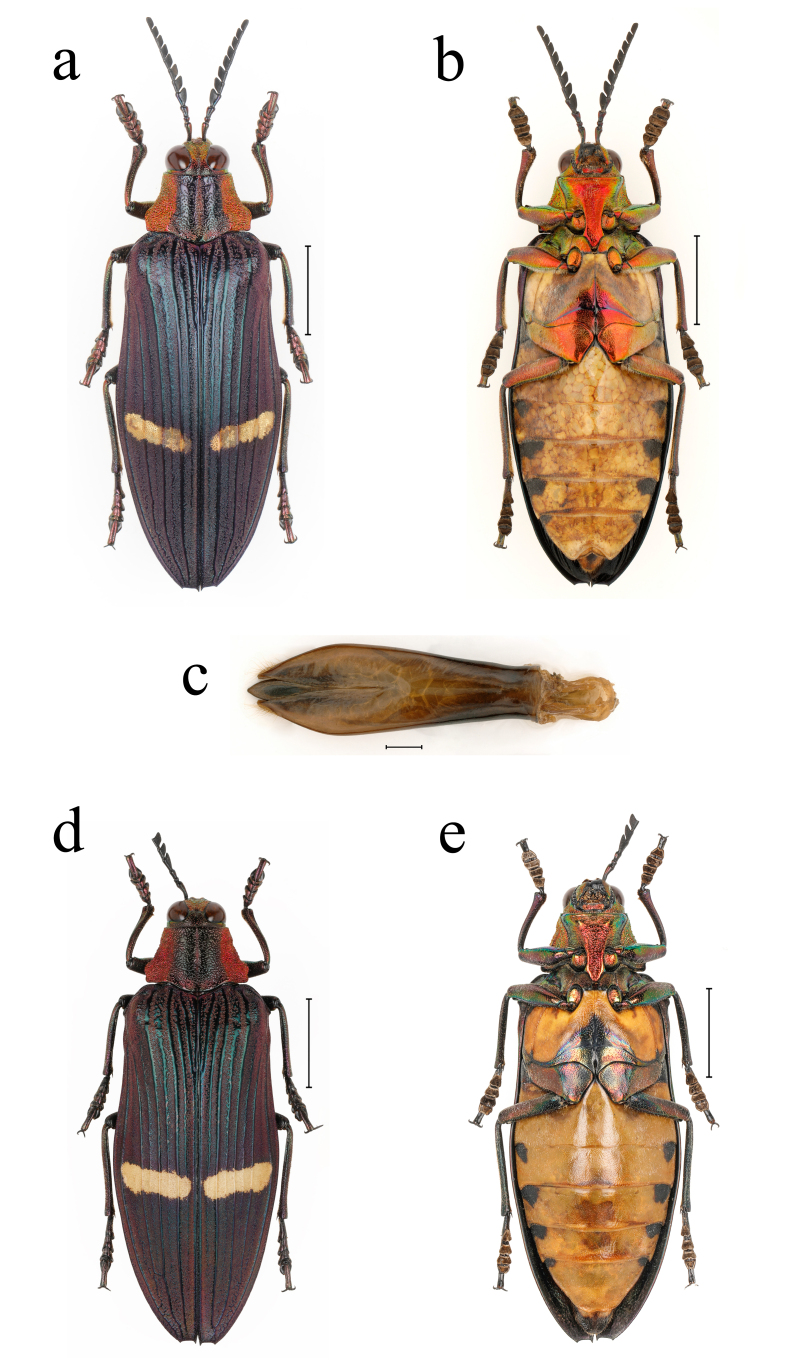
*Xanthocatabonvouloirii* (Deyrolle, 1861) from Yingjiang, Yunnan Province: **a** male dorsal view; **b** male ventral view; **c** dorsal view of aedeagus; **d** female dorsal view; **e** female ventral view. Scale bars: a, b, d, e = 10.0 mm; c = 1.0 mm.

**Description**:

**Male** (Fig. 6): Body (n = 1) length: 51.1–51.5 mm, width: 18.2–19.1 mm, length/width ratio: 2.67–2.83. Body trichromatic and moderately shiny; head, pronotum and elytra densely punctate. Head tricoloured, metallic green to violet, frons with an obviously longitudinal depression along the middle. Pronotum transverse, subtrapezoidal; bicoloured, metallic orange-red with two dark purple longitudinal stripes on both sides of middle line; lateral margins protruding strongly at half of the base, widest behind the middle. Scutellum absent. Elytra bicoloured, dark violet to black with two large, transverse, white stripes behind the middle; widest at nearly half of elytra, each elytron with eight convex costae; lateral margins of elytra smooth, non-serrated, apices of elytra nearly truncate with two small spines at apex of each elytron. All tibiae and femora metallic green to dark purple; outer margin at apex of protibia with nearly triangular protrusion, inner margin at apex densely setose. Ventrites yellow, non-metallic lustre, each ventrite with two large black spots at both sides of the base, ventrite V triangularly excavated at tip. The characters of the male genitalia are shown in Fig. 6.

**Female** (Fig. 6): Body (n = 1) length: 51.6 mm, width: 19.1 mm, length/width ratio: 2.70. Ventrite V without triangular excavation at tip. Other morphological descriptions refer to male specimens.

#### Dicercini

Gistel, 1848

D68A3671-0962-5B3F-8EC9-294CC2C330B9

#### Cardiaspis

E. Saunders, 1866

F65B9FB4-855F-5C7E-BCE5-DD9B14A76338

##### Notes

New record of China.

#### Cardiaspis mouhotii

E. Saunders, 1866

161B6E21-0839-52E8-B668-156685E6B785

Cardiaspis mouhotii E. Saunders, 1866: 307. Saunders 1866

##### Materials

- **Type status:** Other material. **Occurrence:** individualCount: 1; sex: female; lifeStage: adult; occurrenceID: E260F3C5-F7F6-535D-9943-9AE276D4B0CF; **Location:** country: China; stateProvince: Yunnan; locationRemarks: label: Yunnan, Xishuangbanna Dai Autonomous Prefecture, Jinghong City, Mengyang Town, 01.09.2020, Zhong-Xiong Fu Leg.; **Identification:** identifiedBy: Hai-Tian Song; dateIdentified: 2020; **Event:** habitat: Rain Forest; **Record Level:** institutionCode: CHTS; basisOfRecord: PreservedSpecimen
- **Type status:** Other material. **Occurrence:** sex: female; lifeStage: adult; occurrenceID: 6AE549FC-CB79-5AE7-9C7B-ABD423870CA9; **Location:** country: China; stateProvince: Yunnan; verbatimElevation: 800 m; locationRemarks: label: Yunnan, Xishuangbanna Dai Autonomous Prefecture, Mengla County, Wangtianshu Scenic Region, 12.07.2022, Pei-Lin Xue Leg.; **Identification:** identifiedBy: Zhi-Hao Qi; dateIdentified: 2022; **Event:** habitat: Rain Forest; **Record Level:** institutionCode: CHTS; basisOfRecord: PreservedSpecimen
- **Type status:** Other material. **Occurrence:** individualCount: 1; sex: female; lifeStage: adult; occurrenceID: 84639BEB-AE46-5BC9-A462-22E2F2CD692F; **Location:** country: China; stateProvince: Yunnan; verbatimElevation: 1456 m; locationRemarks: label: Yunnan, Xishuangbanna Dai Autonomous Prefecture, Jinghong City, Jinuoshan, 24.07.2022 Hai-Tian Song Leg.; verbatimCoordinates: 22°03'N, 100°40'E; **Identification:** identifiedBy: Hai-Tian Song; dateIdentified: 2022; **Event:** samplingProtocol: flight intercept trap; habitat: Rain Forest; **Record Level:** institutionCode: CHTS; basisOfRecord: PreservedSpecimen
- **Type status:** Other material. **Occurrence:** individualCount: 1; sex: female; lifeStage: adult; occurrenceID: 0EB2C03C-5533-5D45-B6AA-C81FAF3DAB0F; **Location:** country: China; stateProvince: Yunnan; verbatimElevation: 1024 m; locationRemarks: label: Yunnan, Xishuangbanna Dai Autonomous Prefecture, Jinghong City, Jinuoshan, 14.09.2022, Hai-Tian Song Leg.; verbatimCoordinates: 22°05'N, 101°00'E; **Identification:** identifiedBy: Hai-Tian Song; dateIdentified: 2022; **Event:** samplingProtocol: flight intercept trap; habitat: Rain Forest; **Record Level:** institutionCode: CHTS; basisOfRecord: PreservedSpecimen
- **Type status:** Other material. **Occurrence:** individualCount: 1; sex: unknowm; lifeStage: adult; occurrenceID: 7488BAAD-FD1D-5382-B9F1-413451F67C2B; **Location:** country: China; stateProvince: Yunnan; locationRemarks: label: Yunnan, Xishuangbanna Dai Autonomous Prefecture, Jinghong City, Xiaomengyang, 30.05.1981, Da-Zhi Dong Leg.; **Identification:** identifiedBy: Hai-Tian Song; dateIdentified: 2021; **Event:** habitat: Rain Forest; **Record Level:** institutionCode: KIZ; basisOfRecord: PreservedSpecimen

##### Distribution

China (Yunnan) (new country record); Bhutan; India; Laos; Malaysia; Vietnam; Thailand (Akiyama and Ohmomo 2000, Bellamy 2008a, Ek-Amnuay 2008, Kubáň 2016).

##### Notes

**Figure 7.**
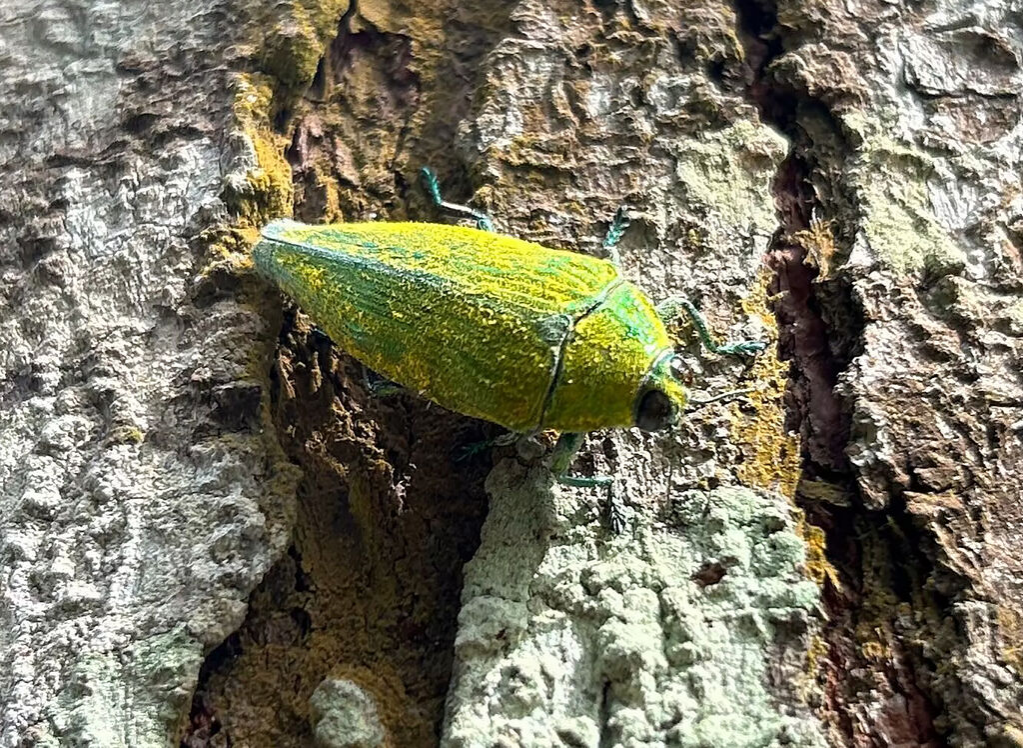
A living individual of *Cardiaspismouhotii* E. Saunders, 1866 from Mengla, Yunnan Province; the photo taken by Yi-Wei Wang in Xishuangbanna Tropical Botanical Garden of Chinese Academy of Sciences on 13 August 2023.

The body surface of the living adult with a yellow wax layer formed by powdery substance (Fig. 7).

**Figure 8.**
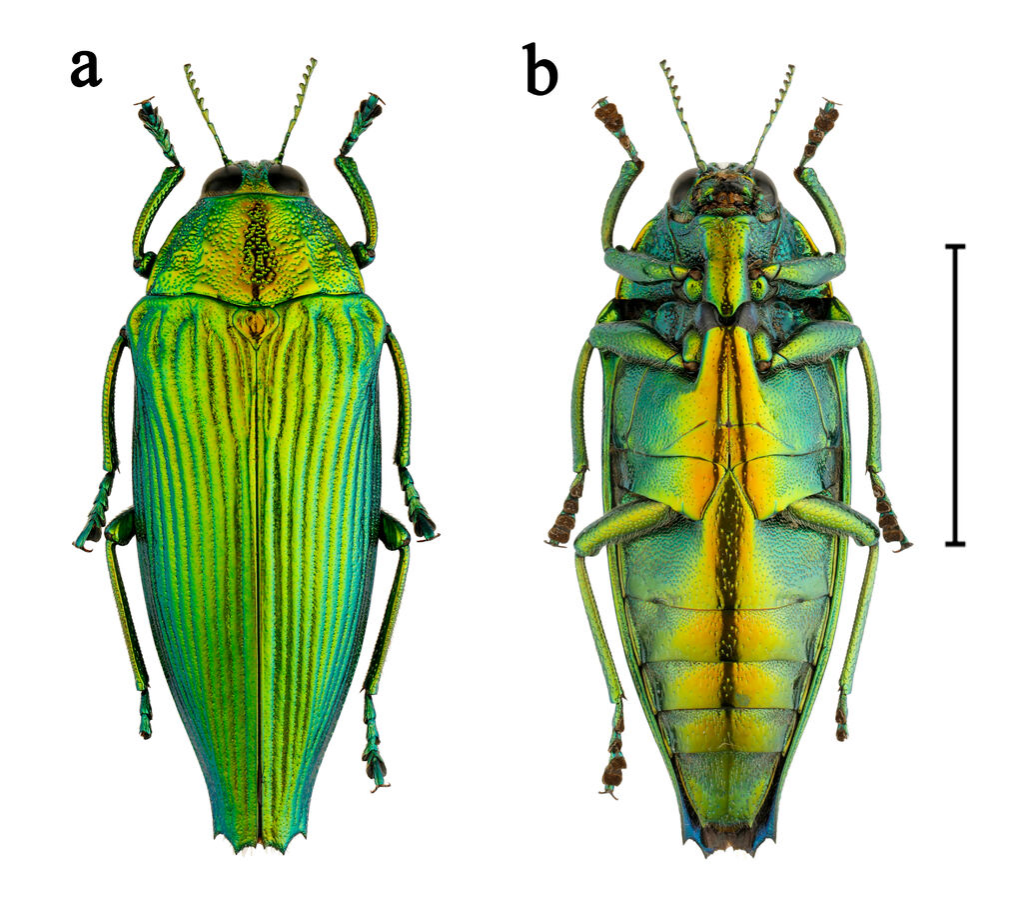
*Cardiaspismouhotii* E. Saunders, 1866, female, from Mengla, Yunnan: **a** dorsal view; **b** ventral view. Scale bar = 10.0 mm.

**Description**:

Female (Fig. 8): Body (n = 4) length: 23.9–29.8 mm, width: 8.9–11.4 mm, length/width ratio: 2.60–2.68. Body monochromatic metallic green with some metallic yellow, shiny; head and pronotum, densely and larger punctate, elytra very small punctate. Head small, frons with two longitudinal carinae along the eyes. Pronotum transverse, subtrapezoidal with lateral depressions moderate; anterior margin almost straight, lateral margins protruding outwards widely, posterior margin bisinuate, widest at base. Scutellum heart-shaped. Each elytron with eleven convex costae; lateral margins of elytra smooth, non-serrated, apices of elytra enlarged with three obvious spines at apex of each elytron. Protibia short, slightly incurved, outer margin with weak serrations, apex without distinct protrusion, inner margin at apex densely setose. Anterior margin of ventrite V protrusion at middle.

**Male**: not examined.

## Discussion

Due to a lack of understanding of their habits, most species of Chrysochroinae from China are often difficult to collect in the wild. It is considered that, especially in south China, there may be more potential species waiting to be discovered. It is hoped that more biological information, such as host plants, distribution and occurrence of the species can be recorded in future studies to provide reference for us to understand the habits of these species.

## Supplementary Material

XML Treatment for Buprestidae

XML Treatment for Chrysochroinae

XML Treatment for Chrysochroini

XML Treatment for Chrysochroa

XML Treatment for Chroodema

XML Treatment for Chrysochroa (Chroodema) corbetti

XML Treatment for Pyranthe

XML Treatment for Chrysochroa (Pyranthe) fulgens ephippigera

XML Treatment for Demochroa

XML Treatment for Demoxantha

XML Treatment for Demochroa (Demoxantha) gratiosa indica

XML Treatment for Xanthocata

XML Treatment for Xanthocata bonvouloirii

XML Treatment for Dicercini

XML Treatment for Cardiaspis

XML Treatment for Cardiaspis mouhotii

## References

[B11219907] Akiyama K., Ohmomo S. (2000). The Buprestid Beetles of the World..

[B11166885] Bellamy C. L. (2008). A world catalogue and bibliography of the jewel beetles (Coleoptera: Buprestoidea). Vol. 1. introduction; fossil taxa; Schizopodidae; Buprestidae: Julodinae—Chrysochroinae: Poecilonotini..

[B11166894] Bellamy C. L. (2008). A World Catalogue and Bibliography of the Jewel Beetles (Coleoptera: Buprestoidea). Vol. 2. Chrysochroinae: Sphenopterini through Buprestinae: Stigmoderini..

[B11213760] Csiki E. (1900). Coleoptera nova in collectione Musei Nationalis Hungarici.. Természetrajzi Füzetek.

[B11189253] Deyrolle H. (1861). Description de deux Buprestides nouveaux (*Catoxantha Bonvouloiri* et *Chrysochroa Mniszechii*). Annales de la Société entomologique de France.

[B10628757] Ek-Amnuay P. (2008). Beetles of Thailand..

[B11166294] Frank David, Sekerka Lukáš (2020). Studies on the genus *Chrysodema* (Coleoptera: Buprestidae: Chrysochroinae) part I. Zootaxa.

[B10628004] Frank D. (2022). Studies on the genus *Chrysodema* (Coleoptera: Buprestidae: Chrysochroinae). Part II.. Zootaxa.

[B11189280] Hołyński R. B. (2009). Taxonomic structure of the subtribe Chrysochroina Cast. with review of the genus *Chrysochroa* Dej. (Coleoptera: Buprestidae).

[B10628566] Hołyński R. B. (2023). A new species of *Chrysochroa* DEJ. (Coleoptera: Buprestidae) from China.. Procrustomachia.

[B11189208] Kerremans C. (1893). Additions aux Buprestides des Indes Orientales. Annales de la Société entomologique de Belgique.

[B10628608] Kubáň V., Löbl I., Löbl D. (2016). Catalogue of Palaearctic Coleoptera. Revised and Updated Edition. Scarabaeoidea, Scirtoidea, Dascilloidea, Buprestoidea, Byrrhoidea..

[B10632508] Ong U., Hattori T. (2019). Jewel beetles of Taiwan.

[B10628537] Ong U., Curletti G., Hattori T. (2023). Jewel beetles of Taiwan Vol. 2: Buprestidae.

[B10627968] Peng Z. L., Shi A. M., Xu H. X., Song H. T. (2021). An Iconography of Chinese Buprestidae (Coleoptera)..

[B10628031] Peng Z. L., Song H. T. (2022). A new species of the genus *Nipponobuprestis* Tôyama, 1986 from Jiangsu, China (Coleoptera: Buprestidae: Chrysochroinae).. The Pan-Pacific Entomologist.

[B10627986] Qi Z. H., Ai H. M., Song H. T. (2022). Notes on the genus *Chrysodema* from China (Coleoptera: Buprestidae: Chrysochroinae), with one new species and one new record.. Zootaxa.

[B11189271] Saunders E. (1866). Catalogue of Buprestidae collected by the late M. Mouhot, in Siam, &c., with descriptions of new species. Transactions of the Entomological Society of London.

[B11189262] Schaufuss L. W. (1863). *Catoxantharegina* n. sp. Sitzungs-Berichte der Naturwissenschaftlichen Gesellschaft Isis zu Dresden.

[B11189244] Waterhouse C. O. (1904). Observations on Coleoptera of the family Buprestidae, with descriptions of new species. The Annals and Magazine of Natural History.

[B11189217] White A. (1843). Descriptions of apparently new species and varieties of insects and other Annulosa, principally from the collection in the British Museum, Coleoptera, Lepidoptera and Crustacea. The Annals and Magazine of Natural History.

